# *PLXNC1*: A Novel Potential Immune-Related Target for Stomach Adenocarcinoma

**DOI:** 10.3389/fcell.2021.662707

**Published:** 2021-07-02

**Authors:** Zhizhan Ni, Chenshen Huang, Hongmei Zhao, Jinzhe Zhou, Muren Hu, Qing Chen, Bujun Ge, Qi Huang

**Affiliations:** ^1^Department of General Surgery, Tongji Hospital, School of Medicine, Tongji University, Shanghai, China; ^2^Department of VIP Clinic, East Hospital, School of Medicine, Tongji University, Shanghai, China; ^3^Department of General Surgery, Shanghai Tenth People’s Hospital, School of Medicine, Tongji University, Shanghai, China

**Keywords:** gastric cancer, plexin C1, tumor microenvironment, macrophage, stomach adenocarcinoma, chromatin accessibility

## Abstract

**Background:**

Gastric cancer is associated with tumor microenvironment and chronic inflammation, but the underlying tumor-promoting mechanisms still remain unknown.

**Methods:**

The ATAC-seq was used to identify genes with chromatin accessibilities in promoter regions. The RNA-seq datasets were performed to identify differentially expressed genes (DEGs). Pearson correlation analysis with the mRNA expression of three families of tumor-related inflammation TFs was used to filter downstream DEGs. Cox univariate survival analysis was performed to identify the prognostic value. The ImmPort database and CIBERSORTx algorithm were used to investigate the regulatory relationship between hub DEGs and immune cells. Immunohistochemistry (IHC) and multidimensional database were performed to verification.

**Results:**

In this case, we require 2,454 genes with chromatin accessibility in promoter regions by ATAC-seq. Based on the gene expression profiles (RNA-seq), we identified 365 genes with chromatin accessibility and differential expression. Combined with the Cox univariate survival analysis, we identified 32 survival-related DEGs with chromatin accessibility. According to ImmPort database, *CXCL3, PLXNC1*, and *EDN2* were identified as immune- related genes in STAD. By applying the CIBERSORTx algorithm and Pearson correlation, *PLXNC1* was the only gene correlated with various immune cells, significantly associated with M2 macrophages. Furthermore, gene set variation analysis (GSVA) suggests the “hallmark_interferon_gamma_response” pathway was most significantly correlated with *PLXNC1.* Immunohistochemistry results revealed that PLXNC1 protein level was significantly higher in STAD tissues than in normal tissues (*p* < 0.001).

**Conclusion:**

*PLXNC1*, regulated by IRF5, is an immune-related gene that was significantly associated with M2 macrophages and poor outcome in stomach adenocarcinoma.

## Introduction

Gastric cancer (GC), the third leading cause of malignancy-related deaths, remains a considerable health problem worldwide (Siegel et al., 2019). Stomach adenocarcinoma (STAD) accounts for approximately 95% of all GC cases (Ajani et al., 2017). Although there have been great advances in diagnostic technology and therapeutic methods in recent decades, the prognosis of STAD patients remains poor.

Chronic inflammation due to infection with *Helicobacter pylori* is associated with GC (Balkwill and Mantovani, 2001). Much attention has been paid to the inflammatory response during initiation, promotion, and progression of GC (Mantovani et al., 2008; Bernard et al., 2019). Not only regarded as a model of inflammation-related cancer, the tumor immune microenvironment plays a vital role in tumor progression, especially tumor-associated macrophages (Li et al., 2019). Accumulating evidence indicates that a high rate of macrophage infiltration is strongly associated with poor prognosis of STAD (Su et al., 2018). Among the variety of molecules involved in tumor-related inflammation, signal transducers and activators of transcription (STATs), nuclear factor kB (NF-kB), and interferon regulatory factors (IRFs) are three crucial families of transcription factors (TFs) that control the inflammatory response (Yu et al., 2009; Yamashita et al., 2010; O’Reilly et al., 2018; Platanitis and Decker, 2018).

Chromatin accessibility maps are vital for the functional interpretation of DNA sequences. ATAC-seq (assay for transposase-accessible chromatin using sequencing), DNase I-seq (DNase I hypersensitive sites sequencing), and NOMe-seq (nucleosome occupancy and methylome sequencing) are the three main experimental methods employed to analyze chromatin accessibility at a genome-wide level. ATAC-seq identifies accessible chromatin regions (Nordstrom et al., 2019), through which enhancers, promoters, insulators, and chromatin-binding factors cooperatively regulate gene expression. ATAC-seq is increasingly used because of its relative technical simplicity and high sensitivity (Picelli et al., 2014; Corces et al., 2016).

Therefore, the goal of the current study was to identify genes with chromatin accessibility in promoter regions that may be directly downstream of TFs regulating STAT, NF-kB, and IRF proteins, using ATAC-seq data downloaded from The Cancer Genome Atlas (TCGA). After identifying differentially expressed genes (DEGs) by RNA-seq, we constructed a univariate cox regression model to explore genes with prognostic value and definite immune-related genes in the ImmPort database. We then performed correlation analysis with tumor infiltrating immune-cell types using CIBERSORTx. The study identified an immune-related gene that interacts with macrophages, providing a novel potential target for STAD treatment.

## Materials and Methods

### Ethics Statement

The studies involving human participants were reviewed and approved by Shanghai Tongji Hospital (2018-LCYJ-005). The patients/participants provided their written informed consent to participate in this study.

### Data Collection

RNA-seq data and corresponding survival information of patients with STAD were downloaded from TCGA^1^, comprising 375 STAD and 32 normal tissue samples. Data from patients who were followed up < 90 days were deleted to minimize the impact of patient death due to non-tumor-related reasons. ATAC-seq profiles of 21 STAD samples were downloaded from TCGA^2^. The list of STAT, NF-kB, and IRF families of TFs was downloaded from The Human Transcription Factors^3^ database. The list of immune-related genes was downloaded from ImmPort database^4^ (Bhattacharya et al., 2018).

### Screening for DEGs

The R package “limma” was used to analyze DEGs between tumor and normal samples in the RNA-seq dataset. For DEG identification, a false discovery rate (FDR)-adjusted *p* < 0.05 and log 2 | fold-change (FC)| > 1 were considered cut-off criteria for genes with significantly altered expression.

### Chromatin Accessibility Analysis

To explore genes with chromatin accessibility, the “karyoploteR,” “ChIPseeker,” and “TxDb. Hsapiens. UCSC. hg38. knownGene” packages in R software were used on the ATAC-seq profile dataset (Huang et al., 2021). Pearson correlation analysis with the mRNA expression of 21 TFs was used to filter downstream DEGs. Then, the lists acquired from Pearson correlation analysis were merged, and duplicated genes were removed.

### Cox and Kaplan-Meier Survival Analysis

Cox univariate survival analysis of DEGs was performed using “survival” package in R with *p* < 0.05 as the threshold. Survival-related DEGs were identified and those with chromatin accessibility were considered hub DEGs. Kaplan-Meier survival analysis was used for further survival analysis.

### Enumerating Tumor-Infiltrating Immune Cells

Hub DEGs were screened from the list of immune-related genes downloaded from the ImmPort database. CIBERSORTx algorithm was used to enumerate the proportions of immune cells. Normalized gene expression data were uploaded to the CIBERSORTx web portal, and the algorithm was run using the LM22 signature for 100 permutations. Cases with a CIBERSORTx output of *p* < 0.05 were considered eligible for further analysis. The Wilcoxon test was used to analyze differences in the composition of immune cells between STAD and normal tissues.

### Correlation Between Gene Expression and Hallmark Pathways

Hallmark gene sets “h.all.v7.2.symbols.gmt,” downloaded from the GSEA database^5^, were used to find signaling pathways involved in STAD occurrence *via* the “GSVA” package in R. Significantly enriched pathways in the hallmark gene sets were determined applying a threshold of *p* < 0.05 and log 2 | FC| ≥ 0.15. Pearson correlation analysis was performed to identify gene co-expression and significantly altered hallmark pathways. The GeneMANIA database^6^ was employed to analyze the gene-gene interaction network and assess the potential interactive functional-association network of *PLXNC1* (Warde-Farley et al., 2010).

### Multiple Database Validation

A variety of online databases were employed to validate our hypothesis. Most methods used to enumerate immune cells were based on a deconvolution algorithm, such as TIMER (Li et al., 2017)^7^, quanTIseq (Nordstrom et al., 2019), and EPIC (Racle et al., 2017)^8^. The GEPIA online database (Tang et al., 2017)^9^ was employed to validate the differential expression of *PLXNC1* and co-expression analysis of *PLXNC1* and macrophage surface markers. The Kaplan-Meier Plotter online database (Nagy et al., 2018)^10^ was used to validate prognostic value. The LinkedOmics database (Vasaikar et al., 2018)^11^ was used to verify the correlation between clinicopathological features and *PLXNC1* expression.

### Quantitative Real-Time Polymerase Chain Reaction Validation

Clinical samples of STAD and paired non-tumor tissues were acquired from 6 patients from Tongji Hospital, Shanghai, China. The quantitative real-time polymerase chain reaction (qPCR) was carried out using SyberGreen (Yeasen, 11200ES03), cDNA and the human-PLXNC1 primers (Forward: AGAGTCCAACCAATCGCATCA, Reverse: AGTCCTGTTCATTACCACGGT). Total RNA was extracted from STAD or non-tumor tissues or cells using the TRIzol reagent. GAPDH served as an internal control. Every sample in each group was detected in triplicate. Paired *t*-test was applied for paired samples in qPCR results.

### Tissue Microarray and Immunohistochemistry

Immunohistochemistry (IHC) was performed on tissue microarray slides (Cancer-cell Tumor Sample Center, Soochow University, Changzhou, China, #20191022R20), comprising 56 tumor and normal tissues. Patient information was provided by the supplier. The slides were deparaffinized in xylene and rehydrated in ethanol. Antigen retrieval was performed using citrate buffer solution (pH = 6.0) with a pressure cooker. Endogenous peroxidases were quenched using 3% H_2_O_2_ for 25 min. Slides were blocked with 3% bovine serum albumin for 30 min at room temperature and then incubated overnight at 4°C in primary antibody anti-PLXNC1 (1:100; Bioss Antibodies, Beijing, China). After the slides were incubated with secondary antibody and stained, hematoxylin counterstaining was used to visualize cell nuclei. Image Pro Plus software (Media Cybernetics, Inc., Rockville, MD, United States) was used to quantify IHC-positive stained cells.

### Statistical Analysis

All statistical analysis were performed with R software version 4.0.2. *P* value < 0.05 and log 2 | fold-change (FC)| > 1 were considered cut-off criteria for genes with significantly altered expression. Significantly enriched pathways in the hallmark gene sets were determined applying a threshold of *p* < 0.05 and log 2 | FC| ≥ 0.15. Paired *t*-test was used for paired samples in qPCR and immunohistochemical analysis.

## Results

### Examining the TF Catalog

A literature search was performed to identify proteins belonging to the STAT, NF-kB, and IRF families of TFs. Then we examined the TF catalog downloaded from The Human Transcription Factors database, comprising 1,639 TF genes (Supplementary Table 1). Among them, the following 21 TFs were examined: STATs 1-4, 5a, 5b, and 6, IRF1-9, NF-kB 1-2, RelA, RelB, and c-Rel. The 21 TFs were used for ATAC-seq analyze.

### Identifying Genes With Chromatin Accessibility

**FIGURE 1:**
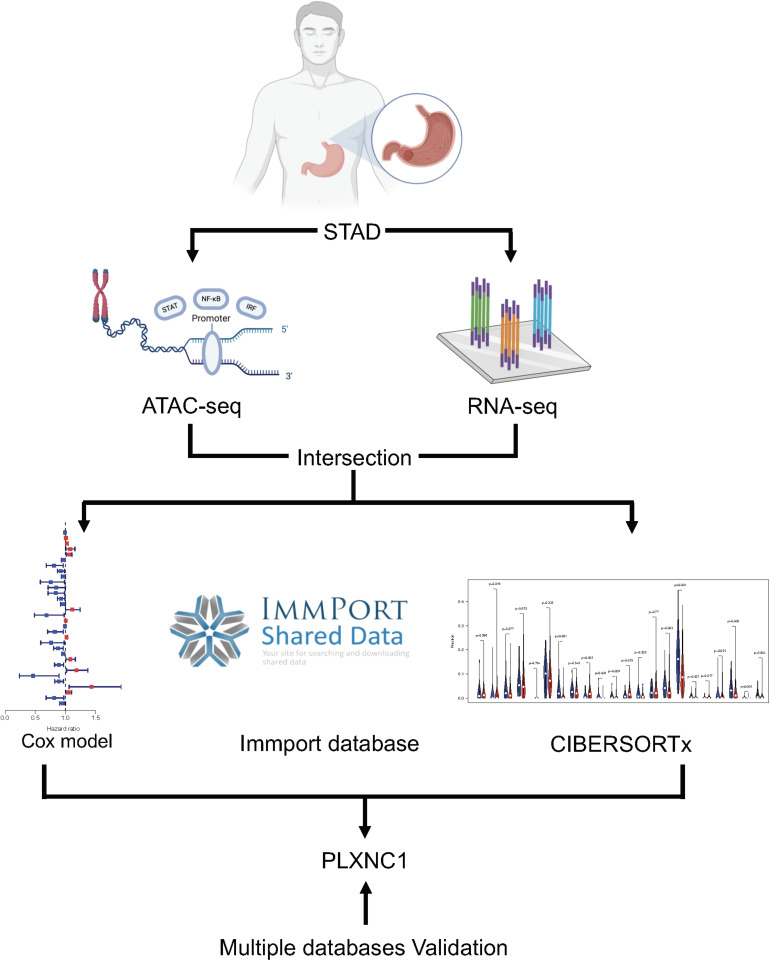
Overview of the study workflow.

**FIGURE 2:**
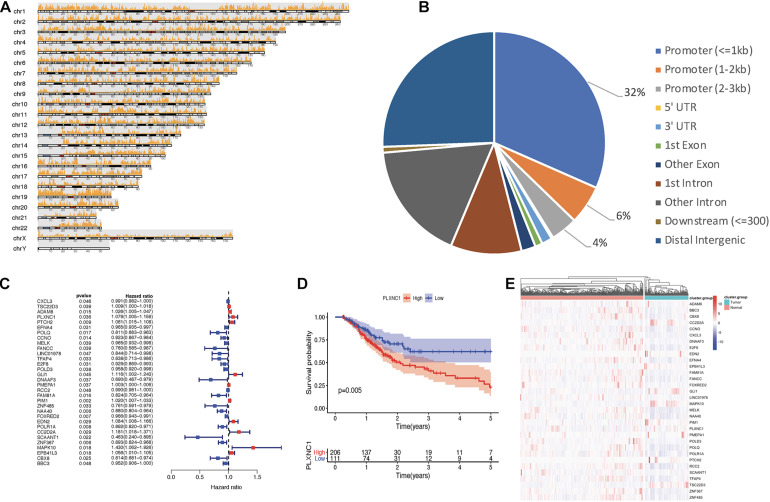
**(A)** Visualization of open regions across the whole genome. **(B)** Pie chart of genomic features indicating that most open chromatin regions were located in promoter regions. **(C)** Forest plot of univariate Cox models. A total of 32 genes were identified as survival-related DEGs, among which 11 DEGs had a hazard ratio (HR) > 1 and 21 DEGs had HR < 1. **(D)** *PLXNC1* was a significant prognostic risk factor for patients with STAD (*p* = 0.005), according to Kaplan-Meier analysis. **(E)** Heatmap for mRNA expression of hub DEGs.

Figure 1 depicts the study workflow. From the ATAC-seq profile dataset, we identified 4,287 genes with chromatin accessibility after removing duplicate genes (Supplementary Table 2). TFs binding to TF-binding sites, especially specific DNA tracts in gene promoter regions, is a central step toward transcriptional repression or activation. Therefore, we searched for genes with chromatin accessibility in these regions, identifying 2,454 genes (Supplementary Table 3). The coverage of chromatin accessibility over the whole genome is shown in Figure 2A while Figure 2B depicts the genomic features.

### Gene Expression and Survival Analyses

A total of 6,739 DEGs were identified in STAD tissues compared to normal adjacent tissues, including 5,593 upregulated and 1,146 downregulated genes. After intersection of the DEGs with chromatin accessibility in promoter regions, we obtained a group of 365 DEGs with chromatin accessibility (Supplementary Table 4). Cox univariate survival analysis was performed, identifying 32 hub DEGs that were significantly related to overall survival of STAD patients (Figure 2C). Then, Kaplan-Meier survival analysis was performed on the hub DEGs (Figure 2D). Figure 2E illustrates the mRNA expression of hub DEGs.

### Correlation Between PLXNC1 and Tumor-Infiltrating Immune Cells

**FIGURE 3:**
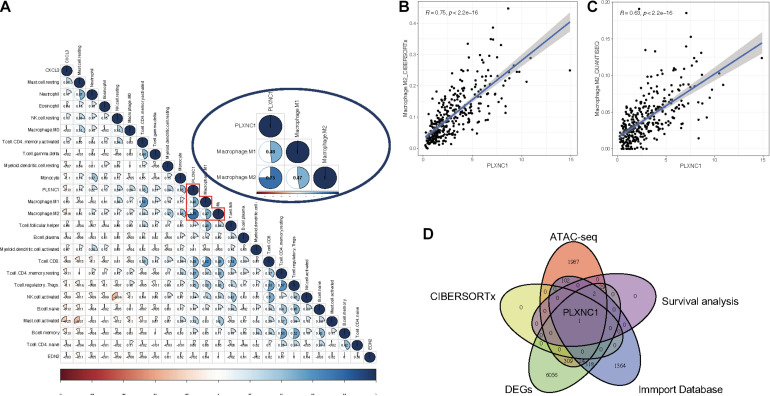
**(A)** Correlation analysis between immune cells and *PLXNC1* expression, which was significantly correlated with M2 macrophages [*r* = 0.75 (Pearson), *p* < 0.01). **(B)** Dot plot demonstrating the correlation between macrophage cells and *PLXNC1* [*r* = 0.75 (Pearson), *p* < 0.01]. **(C)** QUANTISEQ algorithms confirmed the correlation between macrophages and *PLXNC1* [*r* = 0.63 (Pearson), *p* < 0.01]. **(D)** Schematic for co-analysis of the ImmPort database, ATAC-seq, Cox models, RNA-seq and CIBERSORTx in STAD.

A list of 2,498 immune-related genes was downloaded from the ImmPort database, from which the hub DEGs were screened (Supplementary Table 5). The results suggested that *CXCL3, PLXNC1*, and *EDN2* were immune- related genes in STAD. By applying the CIBERSORTx algorithm, the composition of 22 tumor- infiltrating immune cells was acquired for STAD (Supplementary Table 6). To further investigate the regulatory relationship between hub DEGs and immune cells, Pearson correlation analysis was performed (Figure 3A). Among the three genes, *PLXNC1* was the only gene correlated with various immune cells. Furthermore, *PLXNC1* showed the highest correlation with M2 macrophages (*r* = 0.75, *p* < 0.01) (Figure 3B). In order to authenticate the results and minimize bias, other methods (TIMER, quanTIseq, and EPIC) were employed to enumerate immune cells, revealing the same outcome (Figure 3C). Figure 3D visualizes the above-mentioned screening strategy.

### Pathway Enrichment Analysis and Construction of Gene Interaction Network

**FIGURE 4:**
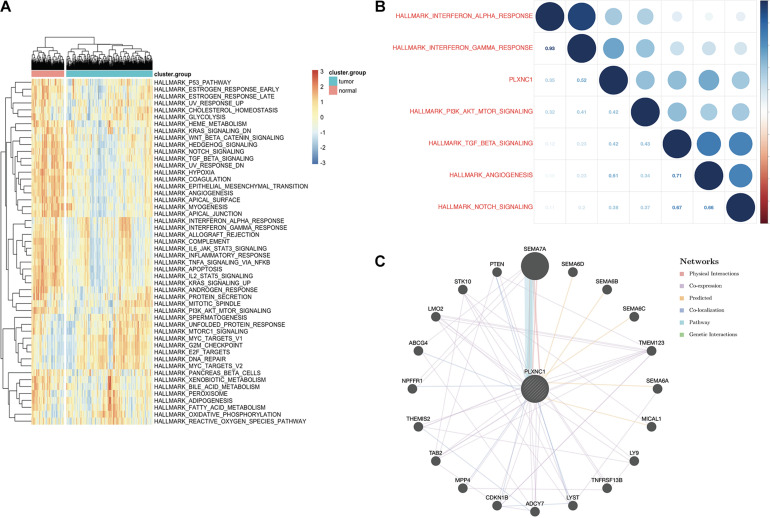
**(A)** Identification of significant pathways correlated with *PLXNC1*. Heatmap displaying 21 significant hallmark pathways differentially expressed in normal and STAD tissues. **(B)** Correlation between the top 5 significant hallmark pathways and *PLXNC1* expression. *PLXNC1* was significantly positively correlated with the “hallmark_interferon_gamma_response” pathway [*r* = 0.75 (Pearson), *p* < 0.01]. **(C)** Gene-gene interaction network analysis of *PLXNC1* and DEGs in the GeneMANIA database. *SEMA7A* most likely interacts with *PLXNC1*.

To explore the upstream of PLXNC1, Pearson correlation analysis between PLXNC1 and 1,639 TF genes at both the mRNA expression level and chromatin accessibility was conducted, respectively. IRF5 was identified the most probably TF of PLXNC1 (Supplementary Tables 7, 8). Gene set variation analysis (GSVA) was performed to identify signaling pathways associated with *PLXNC1* that were significantly up- and downregulated. Four pathways were significantly activated in STAD, while 17 were inhibited (Figure 4A). Pearson analysis was performed to determine correlations between differentially expressed pathways and *PLXNC1*. The “hallmark_interferon_gamma_response” pathway was most significantly correlated with *PLXNC1* (*r* = 0.52, *p* < 0.01) (Figure 4B). The potential interactive functional-association network of *PLXNC1* is shown in Figure 4C.

### Multiple Database Validation

**FIGURE 5:**
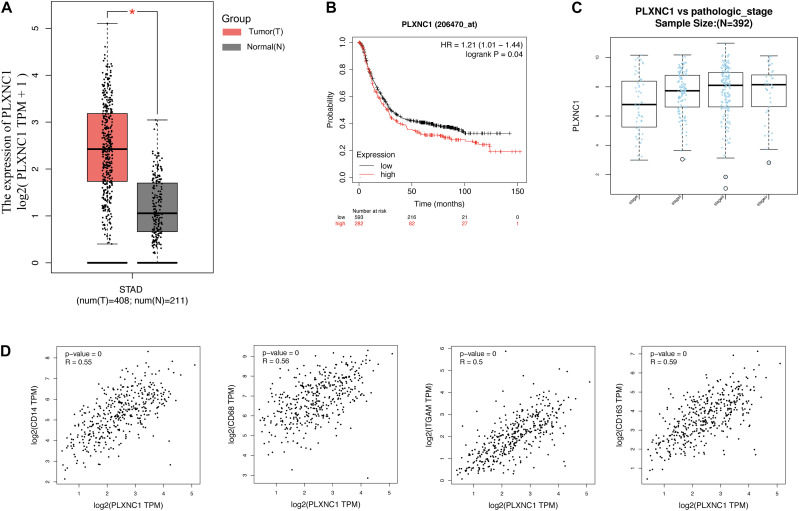
**(A)** *PLXNC1* was significantly upregulated in STAD samples, verified in the GEPIA database with STAD (red) and normal (gray) samples (*p* < 0.05). **(B)** *PLXNC1* was a prognostic risk factor in patients with STAD (log-rank *p* = 0.04) in the Kaplan-Meier Plotter online database. **(C)** *PLXNC1* expression in STAD patients according to pathologic stage in the LinkedOmics database. *PLXNC1* expression increased significantly as pathologic stage progressed (*p* < 0.001). **(D)** Correlation between *PLXNC1* and macrophage surface antigens *CD14* [*r* = 0.56 (Pearson), *p* < 0.0001], *CD68* [*r* = 0.56 (Pearson), *p* < 0.0001], *CD11b* [*r* = 0.5 (Pearson), *p* < 0.0001], and *CD163* [*r* = 0.59 (Pearson), *p* < 0.0001] in the GEPIA database.

Differential expression of *PLXNC1* mRNA in GC was verified in multiple databases. According to the GEPIA database, which included 408 STAD and 211 normal samples, *PLXNC1* expression was significantly higher in STAD tissues than in normal controls (*p* < 0.01) (Figure 5A). The Kaplan-Meier Plotter online database was exploited to verify that higher expression of *PLXNC1* was correlated with poor outcomes in STAD patients (Figure 5B). The LinkedOmics database revealed T stage, histological type, race, number of lymph nodes, and pathologic stage differed significantly in STAD patients according to *PLXNC1* expression (Figure 5C). We also explored correlations between *PLXNC1* and macrophage surface antigen (CD11b, CD68, CD14, and CD163) expression in the GEPIA database (Figure 5D).

### Validation *via* qPCR and Immunohistochemical Analysis

**FIGURE 6:**
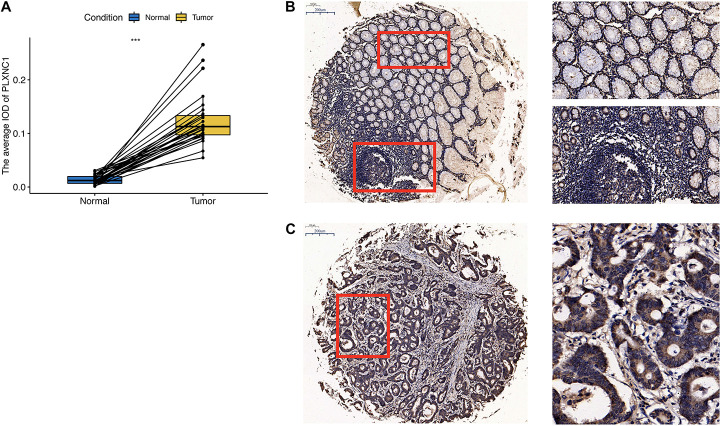
**(A)** PLXNC1 protein expression was significantly upregulated in STAD samples (*N* = 28, paired *t*-test, *p* < 0.001). **(B)** Left image: Immunohistochemical staining examined at ×100 magnification. Red boxes are zoomed in regions corresponding to images on the right. Right images: Low PLXNC1 expression was observed in normal tissues and mainly nuclear staining in a collection of immune cells. The work distance is 0.55, objective lens is 20X Zeiss PlAN-Apo and N.A. is 0.8. **(C)** Immunohistochemical staining of PLXNC1 protein in STAD tissues examined at ×100 magnification. PLXNC1 expression was observed mainly in cytoplasm or membrane staining in tumor cells. The work distance is 0.55, objective lens is 20X Zeiss PlAN-Apo and N.A. is 0.8.

To validate the bioinformatics results, we performed qPCR analysis of STAD tumor and paired non-tumor tissues from five patients with STAD from Shanghai Tongji Hospital. The results were consistent with our bioinformatic findings that the *PLXNC1* expression was significantly upregulated in STAD tissues (*p* = 0.0128, Supplementary Figure 2). Furthermore, we examined PLXNC1 protein expression in STAD tissue microarray samples. As shown in Figure 6A, PLXNC1 protein level was significantly higher in STAD tissues than in normal tissues (*p* < 0.001). Staining of a collection of immune cells and tumor cells can be observed in the picture (Figures 6B–C).

## Discussion

ATAC-seq, widely used for genome-wide identification of open chromatin, is based on the use of hyperactive Tn5 transposase to recognize and cleave DNA in open chromatin regions (Nordstrom et al., 2019). This method requires fewer cells to generate comprehensive maps. The relative simplicity of the protocol makes it a good choice for studies with a large number of samples, especially clinical cohorts. Using ATAC-seq profiles from TCGA and other bioinformatics approaches, we identified 2,454 genes with chromatin accessibility in promoter regions, of which 32 were survival-related DEGs. According to the ImmPort database and deconvolution algorithm validation, *PLXNC1* was significantly associated with macrophages.

*PLXNC1* encodes a member of the plexin family located on chromosome 12, which serves as a receptor for Semaphoring 7A (Sema7A) (Liu et al., 2010). According to The Human Protein Atlas (Uhlen et al., 2015), *PLXNC1* expression is enhanced in macrophages, melanocytes, and Kupffer cells. Semaphorins are a large family of molecular signals regulating cell motility and migration, axon guidance, and the immune response (Zhou et al., 2008). These findings support that *PLXNC1* is associated with the process of inflammation and immune reactions. Furthermore, accumulating evidence has confirmed that *PLXNC1* is aberrantly expressed in a variety of human cancers and serves as either a cancer promoter or suppressor (Wang et al., 2018; Chen et al., 2020). According to the TIMER database (Li et al., 2017), differential expression of *PLXNC1* has been detected in 13 cancer types (CHOL, COAD, ESCA, HNSC, KICH, KIRC, LIHC, LUSC, PRAD, READ, STAD, THCA and UCEC). Among them, *PLXNC1* is highly expressed in CHOL, ESCA, HNSC, KIRC, LIHC, STAD and THCA compared with normal tissues (Supplementary Figure 1). Konig et al. (2014) demonstrated that *PLXNC1* directly influenced critical steps of leukocyte transmigration to promote acute inflammation *in vivo*. In another study, the Sema7A/PlexinC1 interaction was originally shown to induce activation of monocytes (Holmes et al., 2002). It was also found that *PLXNC1* deficiency enabled synaptotagmin 7–mediated macrophage migration and enhanced mammalian lung fibrosis (Peng et al., 2016). When it comes to its role in the multistep cancer process, *PLXNC1* was found to be involved in cancer progression (Balakrishnan et al., 2009). In these studies, *PLXNC1* was suggested to be a protective factor in melanoma, suppressing tumor development. These studies demonstrated that *PLXNC1* participates in the occurrence and development of tumors.

In addition, Chen et al. (2020) were the first researchers to identify that *PLXNC1* was up-regulated in STAD and associated with poor prognosis. They have confirmed that overexpression of *PLXNC1* significantly accelerated carcinogenesis in GC *in vitro* and *in vivo*. Furthermore, *PLXNC1* promoted proliferation and migration of GC cells through transcriptional activation of the interleukin 6 signal transducer (IL6ST).

In our study, *PLXNC1* was significantly correlated with M2 macrophages and most significantly correlated with the “hallmark_interferon_gamma_response” pathway, an immune-related pathway. Therefore, *PLXNC1* may be a key player in the inflammatory and immune responses. Our results also suggested that *PLXNC1* has a prognostic value in STAD, although this was not the emphasis of our study. We focused on the interactions between *PLXNC1* and M2 macrophages during inflammation to enhance STAD proliferation and the regulation of *PLXNC1* by *IRF5*.

Our study has some limitations. First, we did not validate the prognostic value of *PLXNC1* in a clinical cohort, instead we used the Kaplan-Meier Plotter online database for verification. Studies with larger populations and longer follow-up duration will be conducted in the future to confirm our results. Second, our research did not investigate the mechanisms between *PLXNC1* and tumor infiltrating immune cells, especially tumor-infiltrating macrophages. Future studies will focus on these aspects. However, despite the above-mentioned limitations, this is the first study to infer that *PLXNC1* expression might be regulated by *IRF5* and correlated with macrophages in STAD using ATAC-seq and RNA-seq. Molecular biological experiments will be performed to validate these findings in the future.

## Conclusion

Our study demonstrated that *PLXNC1* is regulated by *IRF5*. *PLXNC1* expression was upregulated in patients with STAD and associated with poor outcome. Furthermore, *PLXNC1* was strongly associated with the macrophage fraction. These results reveal that *PLXNC1* is associated with immunity and interacts with macrophages in some manner, suggesting the potential value of anti-PLXNC1 therapy for STAD treatment.

## Data Availability Statement

The original contributions presented in the study are included in the article/Supplementary Material, further inquiries can be directed to the corresponding author/s.

## Ethics Statement

The studies involving human participants were reviewed and approved by Shanghai Tongji Hospital (2018-LCYJ-005). The patients/participants provided their written informed consent to participate in this study.

## Author Contributions

ZN, CH, HZ, QH, and BG conceived the idea and wrote the manuscript. ZN, CH, HZ, JZ, MH, QC, QH, and BG designed the experiments and analyzed the data. All authors read and approved the final manuscript.

## Conflict of Interest

The authors declare that the research was conducted in the absence of any commercial or financial relationships that could be construed as a potential conflict of interest.

## Footnotes

1 https://tcga-data.nci.nih.gov/tcga/

2 https://gdc.cancer.gov/about-data/publications/ATACseq-AW

3 http://humantfs.ccbr.utoronto.ca/

4 https://www.immport.org/shared/home

5 https://www.gsea-msigdb.org/gsea/downloads.jsp

6 http://genemania.org

7 http://timer.comp-genomics.org/

8 https://gfellerlab.shinyapps.io/EPIC_1-1/

9 http://gepia.cancer-pku.cn/

10 https://kmplot.com/analysis/

11 http://www.linkedomics.org/login.php

## Supplementary Material

Supplementary Figure 1 The expression pattern of PLXNC1 in other solid tumors.

Supplementary Figure 2 The relative expression of PLXNC1 mRNA in six patients.

Supplementary Table 1 The result of 1,639 transcription factors genes.

Supplementary Table 2 The result of 4,287 genes with chromatin accessibility after removing duplicate genes.

Supplementary Table 3 The result of 2,454 genes with chromatin accessibility in promoter regions.

Supplementary Table 4 The result of DEGs with chromatin accessibility.

Supplementary Table 5 A list of 2,498 immune-related genes.

Supplementary Table 6 The results of CIBERSORTx in STAD.

Supplementary Table 7 The Pearson correlation analysis between PLXNC1 and 1,639 TF genes at chromatin accessibility.

Supplementary Table 8 The Pearson correlation analysis between PLXNC1 and the top 10 TF genes with chromatin accessibility at mRNA expression level.

Supplementary Table 9 The acronyms are defined in Supplementary Table 9.

## References

[B1] Ajani J. A. Lee J. Sano T. Janjigian Y. Y. Fan D. Song S. (2017). Gastric adenocarcinoma. *Nat. Rev. Dis. Primers* 3:17036. 10.1038/nrdp.2017.36 28569272

[B2] Balakrishnan A. Penachioni J. Y. Lamba S. Bleeker F. E. Zanon C. Rodolfo M. (2009). Molecular profiling of the “plexinome” in melanoma and pancreatic cancer. *Hum. Mutat.* 30 1167–1174. 10.1002/humu.21017 19462467 PMC2989154

[B3] Balkwill F. Mantovani A. (2001). Inflammation and cancer: back to Virchow? *Lancet* 357 539–545. 10.1016/S0140-6736(00)04046-0 11229684

[B4] Bernard V. Semaan A. Huang J. San Lucas F. A. Mulu F. C. Stephens B. M. (2019). Single-cell transcriptomics of pancreatic cancer precursors demonstrates epithelial and microenvironmental heterogeneity as an early event in neoplastic progression. *Clin. Cancer Res.* 25 2194–2205. 10.1158/1078-0432.CCR-18-1955 30385653 PMC6445737

[B5] Bhattacharya S. Dunn P. Thomas C. G. Smith B. Schaefer H. Chen J. (2018). ImmPort, toward repurposing of open access immunological assay data for translational and clinical research. *Sci. Data* 5:180015. 10.1038/sdata.2018.15 29485622 PMC5827693

[B6] Chen J. Liu H. Chen J. Sun B. Wu J. Du C. (2020). PLXNC1 enhances carcinogenesis through transcriptional activation of IL6ST in gastric cancer. *Front. Oncol.* 10:33. 10.3389/fonc.2020.00033 32117710 PMC7010712

[B7] Corces M. R. Buenrostro J. D. Wu B. Greenside P. G. Chan S. M. Koenig J. L. (2016). Lineage-specific and single-cell chromatin accessibility charts human hematopoiesis and leukemia evolution. *Nat. Genet.* 48 1193–1203. 10.1038/ng.3646 27526324 PMC5042844

[B8] Holmes S. Downs A. M. Fosberry A. Hayes P. D. Michalovich D. Murdoch P. (2002). Sema7A is a potent monocyte stimulator. *Scand. J. Immunol.* 56 270–275. 10.1046/j.1365-3083.2002.01129.x 12193228

[B9] Huang C. Huang R. Chen H. Ni Z. Huang Q. Huang Z. (2021). Chromatin accessibility regulates gene expression and correlates with tumor-infiltrating immune cells in gastric adenocarcinoma. *Front. Oncol.* 10:609940. 10.3389/fonc.2020.609940 33469515 PMC7813815

[B10] Konig K. Marth L. Roissant J. Granja T. Jennewein C. Devanathan V. (2014). The plexin C1 receptor promotes acute inflammation. *Eur. J. Immunol.* 44 2648–2658. 10.1002/eji.201343968 24890788

[B11] Li T. Fan J. Wang B. Traugh N. Chen Q. Liu J. S. (2017). TIMER: a web server for comprehensive analysis of tumor-infiltrating immune cells. *Cancer Res.* 77 e108–e110. 10.1158/0008-5472.CAN-17-0307 29092952 PMC6042652

[B12] Li W. Zhang X. Wu F. Zhou Y. Bao Z. Li H. (2019). Gastric cancer-derived mesenchymal stromal cells trigger M2 macrophage polarization that promotes metastasis and EMT in gastric cancer. *Cell Death Dis.* 10:918. 10.1038/s41419-019-2131-y 31801938 PMC6892854

[B13] Liu H. Juo Z. S. Shim A. H. Focia P. J. Chen X. Garcia K. C. (2010). Structural basis of semaphorin-plexin recognition and viral mimicry from Sema7A and A39R complexes with PlexinC1. *Cell* 142 749–761. 10.1016/j.cell.2010.07.040 20727575 PMC2936782

[B14] Mantovani A. Allavena P. Sica A. Balkwill F. (2008). Cancer-related inflammation. *Nature* 454 436–444. 10.1038/nature07205 18650914

[B15] Nagy A. Lanczky A. Menyhart O. Gyorffy B. (2018). Validation of miRNA prognostic power in hepatocellular carcinoma using expression data of independent datasets. *Sci. Rep.* 8:9227. 10.1038/s41598-018-27521-y 29907753 PMC6003936

[B16] Nordstrom K. J. V. Schmidt F. Gasparoni N. Salhab A. Gasparoni G. Kattler K. (2019). Unique and assay specific features of NOMe-, ATAC- and DNase I-seq data. *Nucleic Acids Res.* 47 10580–10596. 10.1093/nar/gkz799 31584093 PMC6847574

[B17] O’Reilly L. A. Putoczki T. L. Mielke L. A. Low J. T. Lin A. Preaudet A. (2018). Loss of NF-kappaB1 causes gastric cancer with aberrant inflammation and expression of immune checkpoint regulators in a STAT-1-dependent manner. *Immunity* 48 570–583.e8. 10.1016/j.immuni.2018.03.003 29562203

[B18] Peng X. Moore M. Mathur A. Zhou Y. Sun H. Gan Y. (2016). Plexin C1 deficiency permits synaptotagmin 7-mediated macrophage migration and enhances mammalian lung fibrosis. *FASEB J.* 30 4056–4070. 10.1096/fj.201600373R 27609773 PMC5102121

[B19] Picelli S. Bjorklund A. K. Reinius B. Sagasser S. Winberg G. Sandberg R. (2014). Tn5 transposase and tagmentation procedures for massively scaled sequencing projects. *Genome Res.* 24 2033–2040. 10.1101/gr.177881.114 25079858 PMC4248319

[B20] Platanitis E. Decker T. (2018). Regulatory networks involving STATs, IRFs, and NFkappaB in inflammation. *Front. Immunol.* 9:2542. 10.3389/fimmu.2018.02542 30483250 PMC6242948

[B21] Racle J. de Jonge K. Baumgaertner P. Speiser D. E. Gfeller D. (2017). Simultaneous enumeration of cancer and immune cell types from bulk tumor gene expression data. *Elife* 6:e26476. 10.7554/eLife.26476 29130882 PMC5718706

[B22] Siegel R. L. Miller K. D. Jemal A. (2019). Cancer statistics, 2019. *CA Cancer J. Clin.* 69 7–34. 10.3322/caac.21551 30620402

[B23] Su C. Y. Fu X. L. Duan W. Yu P. W. Zhao Y. L. (2018). High density of CD68+ tumor-associated macrophages predicts a poor prognosis in gastric cancer mediated by IL-6 expression. *Oncol. Lett.* 15 6217–6224. 10.3892/ol.2018.8119 29616104 PMC5876426

[B24] Tang Z. Li C. Kang B. Gao G. Li C. Zhang Z. (2017). GEPIA: a web server for cancer and normal gene expression profiling and interactive analyses. *Nucleic Acids Res.* 45 W98–W102. 10.1093/nar/gkx247 28407145 PMC5570223

[B25] Uhlen M. Fagerberg L. Hallstrom B. M. Lindskog C. Oksvold P. Mardinoglu A. (2015). Proteomics. tissue-based map of the human proteome. *Science* 347:1260419. 10.1126/science.1260419 25613900

[B26] Vasaikar S. V. Straub P. Wang J. Zhang B. (2018). LinkedOmics: analyzing multi-omics data within and across 32 cancer types. *Nucleic Acids Res.* 46 D956–D963. 10.1093/nar/gkx1090 29136207 PMC5753188

[B27] Wang Z. Wang X. Zhou H. Dan X. Jiang L. Wu Y. (2018). Long non-coding RNA CASC2 inhibits tumorigenesis via the miR-181a/PLXNC1 axis in melanoma. *Acta Biochim. Biophys. Sin. (Shanghai)* 50 263–272. 10.1093/abbs/gmx148 29514220

[B28] Warde-Farley D. Donaldson S. L. Comes O. Zuberi K. Badrawi R. Chao P. (2010). The GeneMANIA prediction server: biological network integration for gene prioritization and predicting gene function. *Nucleic Acids Res.* 38 W214–W220. 10.1093/nar/gkq537 20576703 PMC2896186

[B29] Yamashita M. Toyota M. Suzuki H. Nojima M. Yamamoto E. Kamimae S. (2010). DNA methylation of interferon regulatory factors in gastric cancer and noncancerous gastric mucosae. *Cancer Sci.* 101 1708–1716. 10.1111/j.1349-7006.2010.01581.x 20507321 PMC11158968

[B30] Yu H. Pardoll D. Jove R. (2009). STATs in cancer inflammation and immunity: a leading role for STAT3. *Nat. Rev. Cancer* 9 798–809. 10.1038/nrc2734 19851315 PMC4856025

[B31] Zhou Y. Gunput R. A. Pasterkamp R. J. (2008). Semaphorin signaling: progress made and promises ahead. *Trends Biochem Sci.* 33 161–170. 10.1016/j.tibs.2008.01.006 18374575

