# Supplementary figures and images for: PLXNC1: A Novel Potential Immune-Related Target for Stomach Adenocarcinoma
Source: Front Cell Dev Biol. 2021 Jul 2;9:662707. doi: 10.3389/fcell.2021.662707 (PMC8283001; doi:10.3389/fcell.2021.662707)

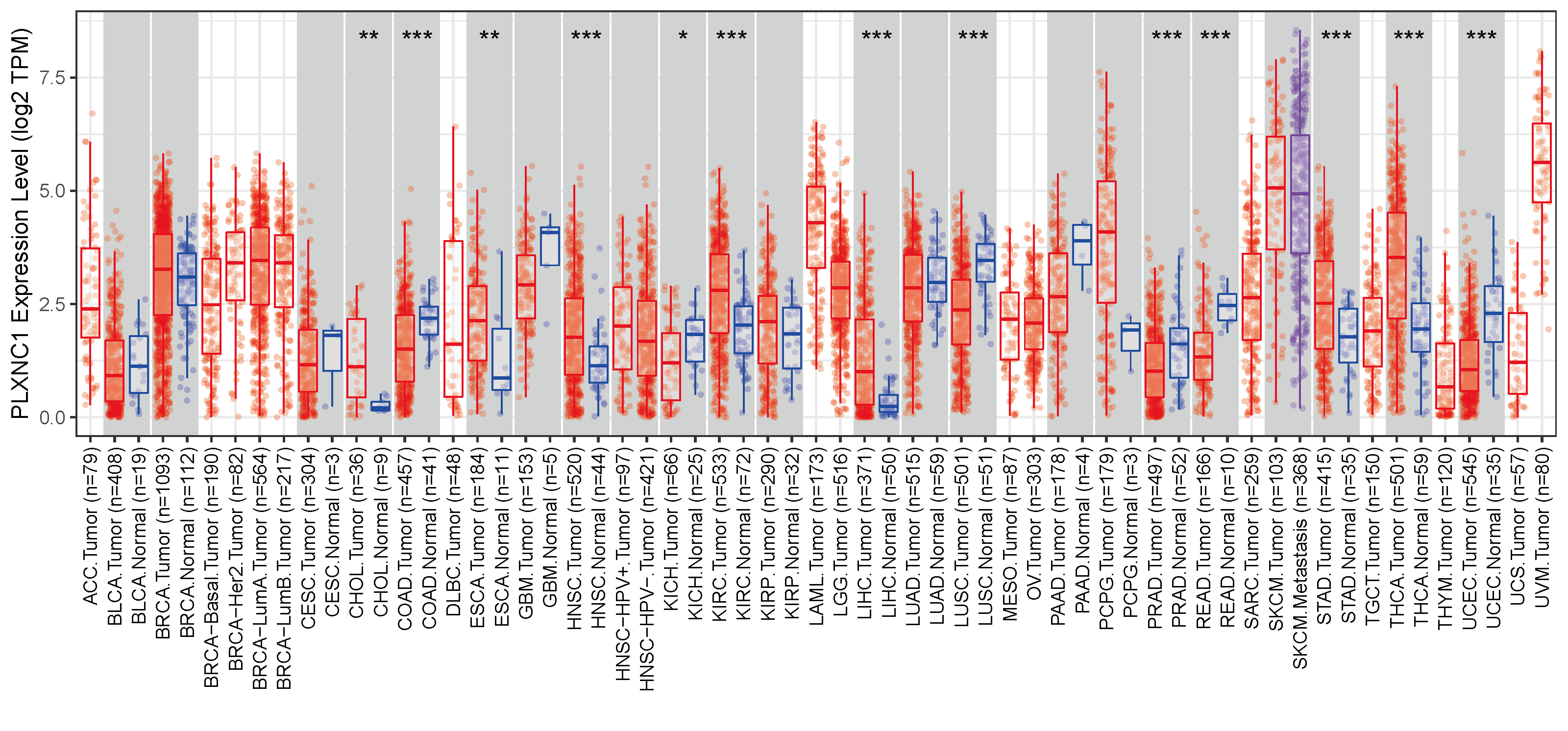

Supplement: Supplementary Figure 1 — The expression pattern of PLXNC1 in other solid tumors. [file Image_1.TIFF]

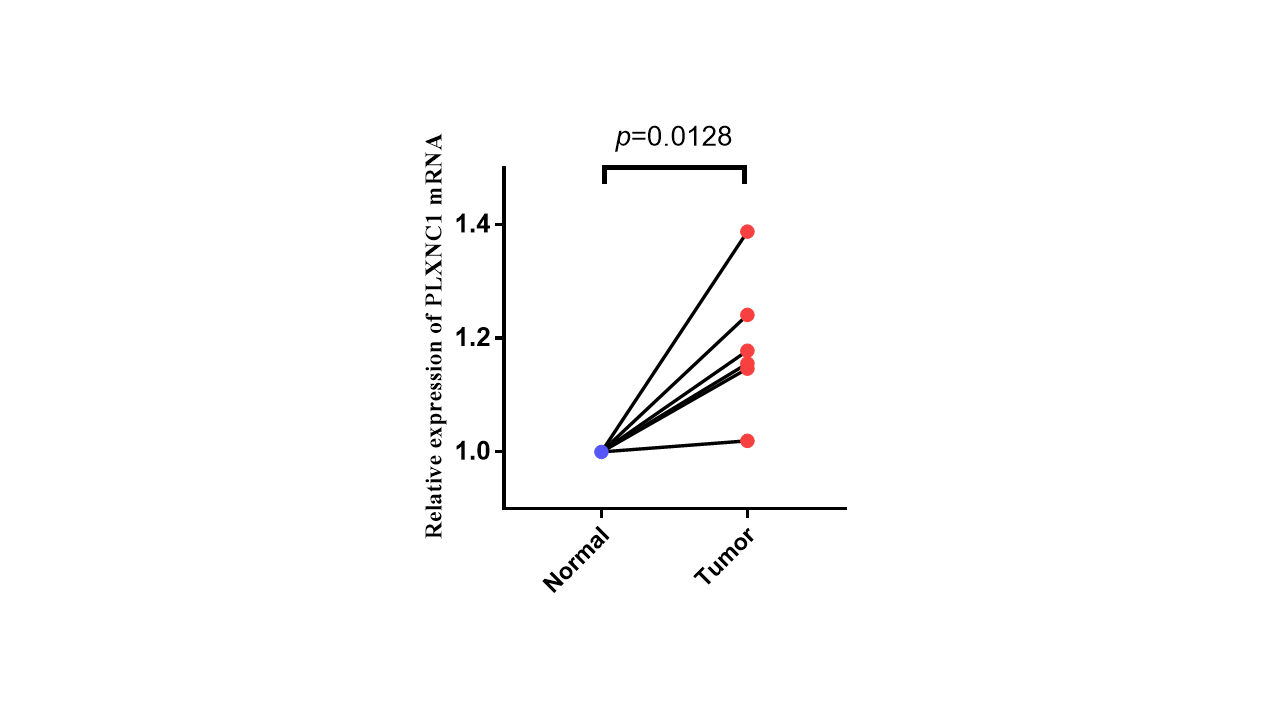

Supplement: Supplementary Figure 2 — The relative expression of PLXNC1 mRNA in six patients. [file Image_2.TIF]
