# Supplementary material for: PLXNC1: A Novel Potential Immune-Related Target for Stomach Adenocarcinoma
Source: Front Cell Dev Biol. 2021 Jul 2;9:662707. doi: 10.3389/fcell.2021.662707 (PMC8283001; doi:10.3389/fcell.2021.662707)
Supplement: Supplementary Table 1 — The result of 1,639 transcription factors genes. [file Table_1.DOCX]

AC008770.3

AC023509.3

AC092835.1

AC138696.1

ADNP

ADNP2

AEBP1

AEBP2

AHCTF1

AHDC1

AHR

AHRR

AIRE

AKAP8

AKAP8L

AKNA

ALX1

ALX3

ALX4

ANHX

ANKZF1

AR

ARGFX

ARHGAP35

ARID2

ARID3A

ARID3B

ARID3C

ARID5A

ARID5B

ARNT

ARNT2

ARNTL

ARNTL2

ARX

ASCL1

ASCL2

ASCL3

ASCL4

ASCL5

ASH1L

ATF1

ATF2

ATF3

ATF4

ATF5

ATF6

ATF6B

ATF7

ATMIN

ATOH1

ATOH7

ATOH8

BACH1

BACH2

BARHL1

BARHL2

BARX1

BARX2

BATF

BATF2

BATF3

BAZ2A

BAZ2B

BBX

BCL11A

BCL11B

BCL6

BCL6B

BHLHA15

BHLHA9

BHLHE22

BHLHE23

BHLHE40

BHLHE41

BNC1

BNC2

BORCS8-MEF2B

BPTF

BRF2

BSX

C11orf95

CAMTA1

CAMTA2

CARF

CASZ1

CBX2

CC2D1A

CCDC169-SOHLH2

CCDC17

CDC5L

CDX1

CDX2

CDX4

CEBPA

CEBPB

CEBPD

CEBPE

CEBPG

CEBPZ

CENPA

CENPB

CENPBD1

CENPS

CENPT

CENPX

CGGBP1

CHAMP1

CHCHD3

CIC

CLOCK

CPEB1

CPXCR1

CREB1

CREB3

CREB3L1

CREB3L2

CREB3L3

CREB3L4

CREB5

CREBL2

CREBZF

CREM

CRX

CSRNP1

CSRNP2

CSRNP3

CTCF

CTCFL

CUX1

CUX2

CXXC1

CXXC4

CXXC5

DACH1

DACH2

DBP

DBX1

DBX2

DDIT3

DEAF1

DLX1

DLX2

DLX3

DLX4

DLX5

DLX6

DMBX1

DMRT1

DMRT2

DMRT3

DMRTA1

DMRTA2

DMRTB1

DMRTC2

DMTF1

DNMT1

DNTTIP1

DOT1L

DPF1

DPF3

DPRX

DR1

DRAP1

DRGX

DUX1

DUX3

DUX4

DUXA

DZIP1

E2F1

E2F2

E2F3

E2F4

E2F5

E2F6

E2F7

E2F8

E4F1

EBF1

EBF2

EBF3

EBF4

EEA1

EGR1

EGR2

EGR3

EGR4

EHF

ELF1

ELF2

ELF3

ELF4

ELF5

ELK1

ELK3

ELK4

EMX1

EMX2

EN1

EN2

EOMES

EPAS1

ERF

ERG

ESR1

ESR2

ESRRA

ESRRB

ESRRG

ESX1

ETS1

ETS2

ETV1

ETV2

ETV3

ETV3L

ETV4

ETV5

ETV6

ETV7

EVX1

EVX2

FAM170A

FAM200B

FBXL19

FERD3L

FEV

FEZF1

FEZF2

FIGLA

FIZ1

FLI1

FLYWCH1

FOS

FOSB

FOSL1

FOSL2

FOXA1

FOXA2

FOXA3

FOXB1

FOXB2

FOXC1

FOXC2

FOXD1

FOXD2

FOXD3

FOXD4

FOXD4L1

FOXD4L3

FOXD4L4

FOXD4L5

FOXD4L6

FOXE1

FOXE3

FOXF1

FOXF2

FOXG1

FOXH1

FOXI1

FOXI2

FOXI3

FOXJ1

FOXJ2

FOXJ3

FOXK1

FOXK2

FOXL1

FOXL2

FOXM1

FOXN1

FOXN2

FOXN3

FOXN4

FOXO1

FOXO3

FOXO4

FOXO6

FOXP1

FOXP2

FOXP3

FOXP4

FOXQ1

FOXR1

FOXR2

FOXS1

GABPA

GATA1

GATA2

GATA3

GATA4

GATA5

GATA6

GATAD2A

GATAD2B

GBX1

GBX2

GCM1

GCM2

GFI1

GFI1B

GLI1

GLI2

GLI3

GLI4

GLIS1

GLIS2

GLIS3

GLMP

GLYR1

GMEB1

GMEB2

GPBP1

GPBP1L1

GRHL1

GRHL2

GRHL3

GSC

GSC2

GSX1

GSX2

GTF2B

GTF2I

GTF2IRD1

GTF2IRD2

GTF2IRD2B

GTF3A

GZF1

HAND1

HAND2

HBP1

HDX

HELT

HES1

HES2

HES3

HES4

HES5

HES6

HES7

HESX1

HEY1

HEY2

HEYL

HHEX

HIC1

HIC2

HIF1A

HIF3A

HINFP

HIVEP1

HIVEP2

HIVEP3

HKR1

HLF

HLX

HMBOX1

HMG20A

HMG20B

HMGA1

HMGA2

HMGN3

HMX1

HMX2

HMX3

HNF1A

HNF1B

HNF4A

HNF4G

HOMEZ

HOXA1

HOXA10

HOXA11

HOXA13

HOXA2

HOXA3

HOXA4

HOXA5

HOXA6

HOXA7

HOXA9

HOXB1

HOXB13

HOXB2

HOXB3

HOXB4

HOXB5

HOXB6

HOXB7

HOXB8

HOXB9

HOXC10

HOXC11

HOXC12

HOXC13

HOXC4

HOXC5

HOXC6

HOXC8

HOXC9

HOXD1

HOXD10

HOXD11

HOXD12

HOXD13

HOXD3

HOXD4

HOXD8

HOXD9

HSF1

HSF2

HSF4

HSF5

HSFX1

HSFX2

HSFY1

HSFY2

IKZF1

IKZF2

IKZF3

IKZF4

IKZF5

INSM1

INSM2

IRF1

IRF2

IRF3

IRF4

IRF5

IRF6

IRF7

IRF8

IRF9

IRX1

IRX2

IRX3

IRX4

IRX5

IRX6

ISL1

ISL2

ISX

JAZF1

JDP2

JRK

JRKL

JUN

JUNB

JUND

KAT7

KCMF1

KCNIP3

KDM2A

KDM2B

KDM5B

KIN

KLF1

KLF10

KLF11

KLF12

KLF13

KLF14

KLF15

KLF16

KLF17

KLF2

KLF3

KLF4

KLF5

KLF6

KLF7

KLF8

KLF9

KMT2A

KMT2B

L3MBTL1

L3MBTL3

L3MBTL4

LBX1

LBX2

LCOR

LCORL

LEF1

LEUTX

LHX1

LHX2

LHX3

LHX4

LHX5

LHX6

LHX8

LHX9

LIN28A

LIN28B

LIN54

LMX1A

LMX1B

LTF

LYL1

MAF

MAFA

MAFB

MAFF

MAFG

MAFK

MAX

MAZ

MBD1

MBD2

MBD3

MBD4

MBD6

MBNL2

MECOM

MECP2

MEF2A

MEF2B

MEF2C

MEF2D

MEIS1

MEIS2

MEIS3

MEOX1

MEOX2

MESP1

MESP2

MGA

MITF

MIXL1

MKX

MLX

MLXIP

MLXIPL

MNT

MNX1

MSANTD1

MSANTD3

MSANTD4

MSC

MSGN1

MSX1

MSX2

MTERF1

MTERF2

MTERF3

MTERF4

MTF1

MTF2

MXD1

MXD3

MXD4

MXI1

MYB

MYBL1

MYBL2

MYC

MYCL

MYCN

MYF5

MYF6

MYNN

MYOD1

MYOG

MYPOP

MYRF

MYRFL

MYSM1

MYT1

MYT1L

MZF1

NACC2

NAIF1

NANOG

NANOGNB

NANOGP8

NCOA1

NCOA2

NCOA3

NEUROD1

NEUROD2

NEUROD4

NEUROD6

NEUROG1

NEUROG2

NEUROG3

NFAT5

NFATC1

NFATC2

NFATC3

NFATC4

NFE2

NFE2L1

NFE2L2

NFE2L3

NFE4

NFIA

NFIB

NFIC

NFIL3

NFIX

NFKB1

NFKB2

NFX1

NFXL1

NFYA

NFYB

NFYC

NHLH1

NHLH2

NKRF

NKX1-1

NKX1-2

NKX2-1

NKX2-2

NKX2-3

NKX2-4

NKX2-5

NKX2-6

NKX2-8

NKX3-1

NKX3-2

NKX6-1

NKX6-2

NKX6-3

NME2

NOBOX

NOTO

NPAS1

NPAS2

NPAS3

NPAS4

NR0B1

NR1D1

NR1D2

NR1H2

NR1H3

NR1H4

NR1I2

NR1I3

NR2C1

NR2C2

NR2E1

NR2E3

NR2F1

NR2F2

NR2F6

NR3C1

NR3C2

NR4A1

NR4A2

NR4A3

NR5A1

NR5A2

NR6A1

NRF1

NRL

OLIG1

OLIG2

OLIG3

ONECUT1

ONECUT2

ONECUT3

OSR1

OSR2

OTP

OTX1

OTX2

OVOL1

OVOL2

OVOL3

PA2G4

PATZ1

PAX1

PAX2

PAX3

PAX4

PAX5

PAX6

PAX7

PAX8

PAX9

PBX1

PBX2

PBX3

PBX4

PCGF2

PCGF6

PDX1

PEG3

PGR

PHF1

PHF19

PHF20

PHF21A

PHOX2A

PHOX2B

PIN1

PITX1

PITX2

PITX3

PKNOX1

PKNOX2

PLAG1

PLAGL1

PLAGL2

PLSCR1

POGK

POU1F1

POU2AF1

POU2F1

POU2F2

POU2F3

POU3F1

POU3F2

POU3F3

POU3F4

POU4F1

POU4F2

POU4F3

POU5F1

POU5F1B

POU5F2

POU6F1

POU6F2

PPARA

PPARD

PPARG

PRDM1

PRDM10

PRDM12

PRDM13

PRDM14

PRDM15

PRDM16

PRDM2

PRDM4

PRDM5

PRDM6

PRDM8

PRDM9

PREB

PRMT3

PROP1

PROX1

PROX2

PRR12

PRRX1

PRRX2

PTF1A

PURA

PURB

PURG

RAG1

RARA

RARB

RARG

RAX

RAX2

RBAK

RBCK1

RBPJ

RBPJL

RBSN

REL

RELA

RELB

REPIN1

REST

REXO4

RFX1

RFX2

RFX3

RFX4

RFX5

RFX6

RFX7

RFX8

RHOXF1

RHOXF2

RHOXF2B

RLF

RORA

RORB

RORC

RREB1

RUNX1

RUNX2

RUNX3

RXRA

RXRB

RXRG

SAFB

SAFB2

SALL1

SALL2

SALL3

SALL4

SATB1

SATB2

SCMH1

SCML4

SCRT1

SCRT2

SCX

SEBOX

SETBP1

SETDB1

SETDB2

SGSM2

SHOX

SHOX2

SIM1

SIM2

SIX1

SIX2

SIX3

SIX4

SIX5

SIX6

SKI

SKIL

SKOR1

SKOR2

SLC2A4RG

SMAD1

SMAD3

SMAD4

SMAD5

SMAD9

SMYD3

SNAI1

SNAI2

SNAI3

SNAPC2

SNAPC4

SNAPC5

SOHLH1

SOHLH2

SON

SOX1

SOX10

SOX11

SOX12

SOX13

SOX14

SOX15

SOX17

SOX18

SOX2

SOX21

SOX3

SOX30

SOX4

SOX5

SOX6

SOX7

SOX8

SOX9

SP1

SP100

SP110

SP140

SP140L

SP2

SP3

SP4

SP5

SP6

SP7

SP8

SP9

SPDEF

SPEN

SPI1

SPIB

SPIC

SPZ1

SRCAP

SREBF1

SREBF2

SRF

SRY

ST18

STAT1

STAT2

STAT3

STAT4

STAT5A

STAT5B

STAT6

T

TAL1

TAL2

TBP

TBPL1

TBPL2

TBR1

TBX1

TBX10

TBX15

TBX18

TBX19

TBX2

TBX20

TBX21

TBX22

TBX3

TBX4

TBX5

TBX6

TCF12

TCF15

TCF20

TCF21

TCF23

TCF24

TCF3

TCF4

TCF7

TCF7L1

TCF7L2

TCFL5

TEAD1

TEAD2

TEAD3

TEAD4

TEF

TERB1

TERF1

TERF2

TET1

TET2

TET3

TFAP2A

TFAP2B

TFAP2C

TFAP2D

TFAP2E

TFAP4

TFCP2

TFCP2L1

TFDP1

TFDP2

TFDP3

TFE3

TFEB

TFEC

TGIF1

TGIF2

TGIF2LX

TGIF2LY

THAP1

THAP10

THAP11

THAP12

THAP2

THAP3

THAP4

THAP5

THAP6

THAP7

THAP8

THAP9

THRA

THRB

THYN1

TIGD1

TIGD2

TIGD3

TIGD4

TIGD5

TIGD6

TIGD7

TLX1

TLX2

TLX3

TMF1

TOPORS

TP53

TP63

TP73

TPRX1

TRAFD1

TRERF1

TRPS1

TSC22D1

TSHZ1

TSHZ2

TSHZ3

TTF1

TWIST1

TWIST2

UBP1

UNCX

USF1

USF2

USF3

VAX1

VAX2

VDR

VENTX

VEZF1

VSX1

VSX2

WIZ

WT1

XBP1

XPA

YBX1

YBX2

YBX3

YY1

YY2

ZBED1

ZBED2

ZBED3

ZBED4

ZBED5

ZBED6

ZBED9

ZBTB1

ZBTB10

ZBTB11

ZBTB12

ZBTB14

ZBTB16

ZBTB17

ZBTB18

ZBTB2

ZBTB20

ZBTB21

ZBTB22

ZBTB24

ZBTB25

ZBTB26

ZBTB3

ZBTB32

ZBTB33

ZBTB34

ZBTB37

ZBTB38

ZBTB39

ZBTB4

ZBTB40

ZBTB41

ZBTB42

ZBTB43

ZBTB44

ZBTB45

ZBTB46

ZBTB47

ZBTB48

ZBTB49

ZBTB5

ZBTB6

ZBTB7A

ZBTB7B

ZBTB7C

ZBTB8A

ZBTB8B

ZBTB9

ZC3H8

ZEB1

ZEB2

ZFAT

ZFHX2

ZFHX3

ZFHX4

ZFP1

ZFP14

ZFP2

ZFP28

ZFP3

ZFP30

ZFP37

ZFP41

ZFP42

ZFP57

ZFP62

ZFP64

ZFP69

ZFP69B

ZFP82

ZFP90

ZFP91

ZFP92

ZFPM1

ZFPM2

ZFX

ZFY

ZGLP1

ZGPAT

ZHX1

ZHX2

ZHX3

ZIC1

ZIC2

ZIC3

ZIC4

ZIC5

ZIK1

ZIM2

ZIM3

ZKSCAN1

ZKSCAN2

ZKSCAN3

ZKSCAN4

ZKSCAN5

ZKSCAN7

ZKSCAN8

ZMAT1

ZMAT4

ZNF10

ZNF100

ZNF101

ZNF107

ZNF112

ZNF114

ZNF117

ZNF12

ZNF121

ZNF124

ZNF131

ZNF132

ZNF133

ZNF134

ZNF135

ZNF136

ZNF138

ZNF14

ZNF140

ZNF141

ZNF142

ZNF143

ZNF146

ZNF148

ZNF154

ZNF155

ZNF157

ZNF16

ZNF160

ZNF165

ZNF169

ZNF17

ZNF174

ZNF175

ZNF177

ZNF18

ZNF180

ZNF181

ZNF182

ZNF184

ZNF189

ZNF19

ZNF195

ZNF197

ZNF2

ZNF20

ZNF200

ZNF202

ZNF205

ZNF207

ZNF208

ZNF211

ZNF212

ZNF213

ZNF214

ZNF215

ZNF217

ZNF219

ZNF22

ZNF221

ZNF222

ZNF223

ZNF224

ZNF225

ZNF226

ZNF227

ZNF229

ZNF23

ZNF230

ZNF232

ZNF233

ZNF234

ZNF235

ZNF236

ZNF239

ZNF24

ZNF248

ZNF25

ZNF250

ZNF251

ZNF253

ZNF254

ZNF256

ZNF257

ZNF26

ZNF260

ZNF263

ZNF264

ZNF266

ZNF267

ZNF268

ZNF273

ZNF274

ZNF275

ZNF276

ZNF277

ZNF28

ZNF280A

ZNF280B

ZNF280C

ZNF280D

ZNF281

ZNF282

ZNF283

ZNF284

ZNF285

ZNF286A

ZNF286B

ZNF287

ZNF292

ZNF296

ZNF3

ZNF30

ZNF300

ZNF302

ZNF304

ZNF311

ZNF316

ZNF317

ZNF318

ZNF319

ZNF32

ZNF320

ZNF322

ZNF324

ZNF324B

ZNF326

ZNF329

ZNF331

ZNF333

ZNF334

ZNF335

ZNF337

ZNF33A

ZNF33B

ZNF34

ZNF341

ZNF343

ZNF345

ZNF346

ZNF347

ZNF35

ZNF350

ZNF354A

ZNF354B

ZNF354C

ZNF358

ZNF362

ZNF365

ZNF366

ZNF367

ZNF37A

ZNF382

ZNF383

ZNF384

ZNF385A

ZNF385B

ZNF385C

ZNF385D

ZNF391

ZNF394

ZNF395

ZNF396

ZNF397

ZNF398

ZNF404

ZNF407

ZNF408

ZNF41

ZNF410

ZNF414

ZNF415

ZNF416

ZNF417

ZNF418

ZNF419

ZNF420

ZNF423

ZNF425

ZNF426

ZNF428

ZNF429

ZNF43

ZNF430

ZNF431

ZNF432

ZNF433

ZNF436

ZNF438

ZNF439

ZNF44

ZNF440

ZNF441

ZNF442

ZNF443

ZNF444

ZNF445

ZNF446

ZNF449

ZNF45

ZNF451

ZNF454

ZNF460

ZNF461

ZNF462

ZNF467

ZNF468

ZNF469

ZNF470

ZNF471

ZNF473

ZNF474

ZNF479

ZNF48

ZNF480

ZNF483

ZNF484

ZNF485

ZNF486

ZNF487

ZNF488

ZNF490

ZNF491

ZNF492

ZNF493

ZNF496

ZNF497

ZNF500

ZNF501

ZNF502

ZNF503

ZNF506

ZNF507

ZNF510

ZNF511

ZNF512

ZNF512B

ZNF513

ZNF514

ZNF516

ZNF517

ZNF518A

ZNF518B

ZNF519

ZNF521

ZNF524

ZNF525

ZNF526

ZNF527

ZNF528

ZNF529

ZNF530

ZNF532

ZNF534

ZNF536

ZNF540

ZNF541

ZNF543

ZNF544

ZNF546

ZNF547

ZNF548

ZNF549

ZNF550

ZNF551

ZNF552

ZNF554

ZNF555

ZNF556

ZNF557

ZNF558

ZNF559

ZNF560

ZNF561

ZNF562

ZNF563

ZNF564

ZNF565

ZNF566

ZNF567

ZNF568

ZNF569

ZNF57

ZNF570

ZNF571

ZNF572

ZNF573

ZNF574

ZNF575

ZNF576

ZNF577

ZNF578

ZNF579

ZNF580

ZNF581

ZNF582

ZNF583

ZNF584

ZNF585A

ZNF585B

ZNF586

ZNF587

ZNF587B

ZNF589

ZNF592

ZNF594

ZNF595

ZNF596

ZNF597

ZNF598

ZNF599

ZNF600

ZNF605

ZNF606

ZNF607

ZNF608

ZNF609

ZNF610

ZNF611

ZNF613

ZNF614

ZNF615

ZNF616

ZNF618

ZNF619

ZNF620

ZNF621

ZNF623

ZNF624

ZNF625

ZNF626

ZNF627

ZNF628

ZNF629

ZNF630

ZNF639

ZNF641

ZNF644

ZNF645

ZNF646

ZNF648

ZNF649

ZNF652

ZNF653

ZNF654

ZNF655

ZNF658

ZNF66

ZNF660

ZNF662

ZNF664

ZNF665

ZNF667

ZNF668

ZNF669

ZNF670

ZNF671

ZNF672

ZNF674

ZNF675

ZNF676

ZNF677

ZNF678

ZNF679

ZNF680

ZNF681

ZNF682

ZNF683

ZNF684

ZNF687

ZNF688

ZNF689

ZNF69

ZNF691

ZNF692

ZNF695

ZNF696

ZNF697

ZNF699

ZNF7

ZNF70

ZNF700

ZNF701

ZNF703

ZNF704

ZNF705A

ZNF705B

ZNF705D

ZNF705E

ZNF705G

ZNF706

ZNF707

ZNF708

ZNF709

ZNF71

ZNF710

ZNF711

ZNF713

ZNF714

ZNF716

ZNF717

ZNF718

ZNF721

ZNF724

ZNF726

ZNF727

ZNF728

ZNF729

ZNF730

ZNF732

ZNF735

ZNF736

ZNF737

ZNF74

ZNF740

ZNF746

ZNF747

ZNF749

ZNF750

ZNF75A

ZNF75D

ZNF76

ZNF761

ZNF763

ZNF764

ZNF765

ZNF766

ZNF768

ZNF77

ZNF770

ZNF771

ZNF772

ZNF773

ZNF774

ZNF775

ZNF776

ZNF777

ZNF778

ZNF780A

ZNF780B

ZNF781

ZNF782

ZNF783

ZNF784

ZNF785

ZNF786

ZNF787

ZNF788

ZNF789

ZNF79

ZNF790

ZNF791

ZNF792

ZNF793

ZNF799

ZNF8

ZNF80

ZNF800

ZNF804A

ZNF804B

ZNF805

ZNF808

ZNF81

ZNF813

ZNF814

ZNF816

ZNF821

ZNF823

ZNF827

ZNF829

ZNF83

ZNF830

ZNF831

ZNF835

ZNF836

ZNF837

ZNF84

ZNF841

ZNF843

ZNF844

ZNF845

ZNF846

ZNF85

ZNF850

ZNF852

ZNF853

ZNF860

ZNF865

ZNF878

ZNF879

ZNF880

ZNF883

ZNF888

ZNF891

ZNF90

ZNF91

ZNF92

ZNF93

ZNF98

ZNF99

ZSCAN1

ZSCAN10

ZSCAN12

ZSCAN16

ZSCAN18

ZSCAN2

ZSCAN20

ZSCAN21

ZSCAN22

ZSCAN23

ZSCAN25

ZSCAN26

ZSCAN29

ZSCAN30

ZSCAN31

ZSCAN32

ZSCAN4

ZSCAN5A

ZSCAN5B

ZSCAN5C

ZSCAN9

ZUFSP

ZXDA

ZXDB

ZXDC

ZZZ3
